# Pairwise and high-order dependencies in the cryptocurrency trading network

**DOI:** 10.1038/s41598-022-21192-6

**Published:** 2022-11-02

**Authors:** Tomas Scagliarini, Giuseppe Pappalardo, Alessio Emanuele Biondo, Alessandro Pluchino, Andrea Rapisarda, Sebastiano Stramaglia

**Affiliations:** 1 Dipartimento Interateneo di Fisica, Università degli Studi Aldo Moro, Bari, Italy; 2 INFN Sezione di Bari, Bari, Italy; 3 Dipartimento di Fisica e Astronomia, Università degli Studi di Catania, Catania, Italy; 4 Dipartimento di Economia e Impresa, Università degli Studi di Catania, Catania, Italy; 5 INFN Sezione di Catania, Catania, Italy; 6 Complexity Science Hub, Vienna, Austria; 7 Center of Innovative Technologies for Signal Detection and Processing (TIRES), Università degli Studi Aldo Moro, Bari, Italy

**Keywords:** Information theory and computation, Complex networks

## Abstract

In this paper we analyse the effects of information flows in cryptocurrency markets. We first define a cryptocurrency trading network, i.e. the network made using cryptocurrencies as nodes and the Granger causality among their weekly log returns as links, later we analyse its evolution over time. In particular, with reference to years 2020 and 2021, we study the logarithmic US dollar price returns of the cryptocurrency trading network using both pairwise and high-order statistical dependencies, quantified by Granger causality and O-information, respectively. With reference to the former, we find that it shows peaks in correspondence of important events, like e.g., Covid-19 pandemic turbulence or occasional sudden prices rise. The corresponding network structure is rather stable, across weekly time windows in the period considered and the coins are the most influential nodes in the network. In the pairwise description of the network, stable coins seem to play a marginal role whereas, turning high-order dependencies, they appear in the highest number of synergistic information circuits, thus proving that they play a major role for high order effects. With reference to redundancy and synergy with the time evolution of the total transactions in US dollars, we find that their large volume in the first semester of 2021 seems to have triggered a transition in the cryptocurrency network toward a more complex dynamical landscape. Our results show that pairwise and high-order descriptions of complex financial systems provide complementary information for cryptocurrency analysis.

## Introduction

The interest in cryptocurrencies increased a lot in the last few years, among investors and researchers. Still recently, until approximately five-seven year ago, the cryptocurrency markets were the object of interest of few professionals, vastly academic, interested on applications, technology and even speculation on DeFi (Decentralized Finance). As data shows, during the year 2017, a huge increase in the USD price of Bitcoin (something like $2300 \%$, from about 800 in January to more than 19000 in December^1^) caused un undoubtedly understandable interest. An even more impressive piece of evidence was that, after just after one month, that same price fell down to 6852 USD - a fall of more than $64 \%$^2^. Also other cryptocurrencies manifested what we could name “turbulence” (at best) but the very peculiar aspect of their dynamics was the impressive volatility, the apparently intrinsic instability of these financial assets.

Needless to say, their markets have become attractive as a very ambitious and dangerous lottery, still able to provide wide returns, at a scale difficultly comparable with “traditional” financial instruments. Since then, a proliferous set of cryptocurrencies has developed and traded on exchanges (ETH since 2015, REP, XRP and ETC since 2016, USDT, XLM and BCH since 2017 among many others, see Table 2 for a fast overview), thus giving rise to an entire “sector”, as if they should behave similarly, all apparently representing the hottest opportunity of finance.

In time, therefore, the predominant position of Bitcoin has diminished and the financial potential of these cryptos is now so fragmented over a numerous set of competing alternatives, that it is very unlikely that one of them can assume any truly monetary role. One of the apparent elements of appeal of cryptos is the absence of a centralized control over them. The basic idea of the blockchain rests more on the side of tracking transactions: encryption prevents any personal identification of market participants and this attracts many forms of traders, investors, negotiators in a wide variety of clear and unclear market transactions. Therefore, the market capitalization of the crypto world has assumed notable dimensions going to $\$14$ billion in 2014 to $\$831$ billion in 2018, still remaining very high after severe reductions in 2018, until 2020 at $\$192$ billion. On the aggregate side, it is easy to collect data of cryptocurrencies, since most of the anonymized exchanges are often publicly available.

In this work, we consider three different classes of cryptocurrencies, according to their different operational architecture: *coins*, having an independent blockchain (a list of records linked using cryptography) and which are usually the most important in terms of market capitalization; *tokens*, i.e. independent projects which use the blockchain of another cryptocoin; *stablecoins*, whose price is held stable with respect to another financial asset, like the USD dollar or other fiat currencies.

Crises show periodically that financial markets are complex systems where interactions among individual participants is much more relevant in terms of dynamics than the individual characteristics of individual participants. One of the most relevant reasons why micro-prudential supervision (e.g., Basel Agreements) is almost vein in order to prevent the systemic risk is that it fails to consider complexity features emerging from interconnectedness among financial institutions and their resulting financial network. Then, given the wild and unpredictable instability of the price of cryptocurrencies, a branch of literature has flourished dealing with analysis of signals and precursors that could be useful to check the existence of correlations and systemic regularities.

In this work, we study the impact of cryptocurrency price returns using statistical tools to detect the flows of information between time series. To this purpose, we first show how, in general, two variables exchange information in a pairwise way by Granger causality, which detects the information flow from a source variable to a target one.

Granger causality is a statistical method aimed to quantify the gain in linear predictability between time series. This definition does not necessarily imply a true causal link between time series, due to the possible presence of confounding or unobserved variables^3^. For this reason, in this context causality must be intended as a form of predictive causality. The idea of defining causality as a useful tool for predictions was introduced by Wiener^4^ and was later implemented by Granger^5,6^ in the context of linear autoregressive models, that will be used in the present work as weight on a directed cryptocurrency traded network where each nodes is a cryptocurrency and each link states if a crypto may be useful on the price prediction of another one. Since 1960, Grange Causality has become a standard tool in econometrics and has recently received a lot of attention in many other contexts, such as neuroscience^7^ and climatology^8^. All these studies take into account the information exchange between pairs of variables, possibly conditioning over a set of confounding elements to remove spurious effects. Recently, it has been shown that in many systems a simple pairwise approach may not fully describe the entire multivariate structure of information exchange, since higher-order effects play an important role in their description^9^.

High-order effects might be appreciated under two points of view: when considering multiplets of variables, the joint effect can be greater than the sum of single contributions, a condition known as *synergy*; alternatively, the case in which the overall effect results lower than the sum of the parts, is called *redundancy*. Stated differently, redundancy occurs when multiple copies of the same information can be found in different parts of the system, while synergy refers to the part of the information that is not stored in any specific element, but rather in the joint state of a group of variables. A trivial example is the case where $X_1$ and $X_2$ are independent binary random variables and $X_3 = \texttt {XOR}(X_1,X_2)$. In this situation, neither $X_1$ nor $X_2$ contain any information about $X_3$; rather, information is completely stored in their joint state.

In a real-world example, the study of how two market indices cooperate to transfer information to a third one has shown non-trivial synergistic effects^10^ using the Partial Information Decomposition, a statistical framework that decomposes information in the unique, redundant and synergistic parts but has the drawback of not being computationally feasible for more than three variables^11^. A simple yet promising approach for quantifying high-order dependencies in a system is the O-information^12^, which quantifies the balance between redundancy and synergy. Speaking qualitatively, in a complex system, the synergy measures its capacity to make integration of information whilst redundancy provides robustness to the system; decomposing interactions between variables into synergistic and redundant components illuminates how the system addresses the trade-off between robustness and integration. For example, recent works focusing on the brain at the macroscale have identified high synergy brain regions which support higher cognitive function^13^ and are affected by the aging process^14^.

Many studies have investigated different aspects of the cryptocurrency time series, from their correlation properties^15,16^ to the possibility of being used for portfolio optimization^17,18^. The spillover effect between the major cryptocurrencies has been investigated by using VAR (Vector Autoregressive Models, as in^19^, for instance), finding that return and volatility spillover is mainly driven by the Bitcoin and this effect was continuously rising during the considered period, suggesting a growing interdependence among cryptocurrencies. Other studies have investigated the relationship among the most important coins using VAR and SVAR (Structural VAR) Granger causality, finding that “Ethereum is likely to be the independent coin in this market, while Bitcoin tends to be the spillover effect recipient”^20^. An example of direct use of Granger Causality test to study information flow between two major cryptocurrencies has been performed^21^, but this study was limited only to Bitcoin and Litecoin. In^22^, multivariate transfer entropy, which is a model-free approach to study information flow^23^, was used to study a network of cryptocurrencies, using a greedy algorithm to detect the most informative drivers. They found an increase in information flow during the market turbulence of March 2020. It has been shown that Granger Causality and transfer entropy are equivalent for Gaussian processes^24^. Being pairwise methods, neither Granger Causality nor Transfer entropy can assess high-order effects. To the best of our knowledge, there are no investigations among cryptocurrencies using high-order dependencies. The present work addresses to contribute in filling this gap, studying a high-order generalization of Granger Causality. We will show that the pairwise approach and the higher-order one give complementary information to describe the cryptocurrency trading network.

The paper is organized as follows. In section Data and section Methods we describe the dataset and the methodology used for the analyses, respectively. In section Results and Discussion we present and discuss the results while, in section Conclusions , we close the paper drawing some conclusions.

## Results and discussion

In this section, we present and discuss the results obtained with both the pairwise Granger causality approach and the O-information methodology, evaluated among the logarithmic returns time series of cryptocurrencies exchanged in US dollars (USD) in the same time window. The number of elements in the data-set at hand increases with time, due to the constant introduction of newer cryptocurrencies in the market. In order to track the qualitative changes in the pattern of information flow, due to a genuine evolution of the system dynamics and not the varying size of the network, all the global information-theoretic quantities are normalized to the size of the system at each time window.

### Pairwise Granger causality

Granger causality (GC) is a statistical method based on linear regression which establishes a directed link between two time series if one, hereafter called source, allows to make better predictions about the future of the other, the target variable, beyond the information contained in the target’s past states. For each one of the 104 weekly windows (from 30 Dec, 2019 to 31 Dec, 2021), Granger causality analysis was conducted for all the $n(n-1)/2$ pair of variables active in that window, which become nodes of the cryptocurrency traded network. For each pair, the optimum model order *p* was chosen using the Bayesian information criterion^25^. A link between a pair of nodes is created only if the GC strength overcomes the significance threshold of $1\%$.

**Figure 1:**
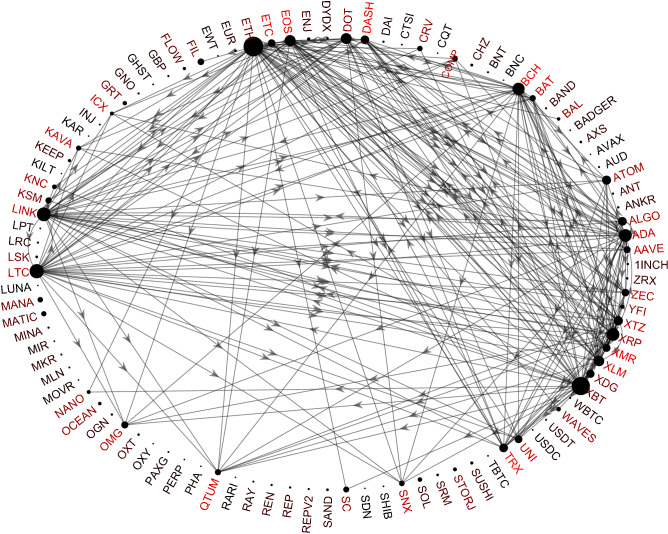
The average USD network of pairwise Granger causality. Each link $\overline{a}_{ij}$ represents the Granger causality averaged over the 104 weekly windows from 28 Dec, 2019 to 30 Dec, 2021. The size of the nodes represents the total out strength $k^{\mathrm{out}}_i = \sum _j \overline{a}_{ij}$, while the label color (from black to red) is the total in strength $k^{\mathrm{in}}_i = \sum _j \overline{a}_{ij}$, that is how much that node is influenced by the rest of the network. A comprehensive list of the most influential and influenced nodes is plotted in Figure 2, where the total out strength and in strength values are shown for each node.

A first visual representation of the structure of the USD network averaged over all the time windows is shown in Fig. 1, where the biggest nodes represent the most influential elements and the color scale of the labels indicates how much a node is influenced by all the others (for a better visualization, only the first $5\%$ of links sorted according to their GC strength is reported in the figure). This kind of graph is very effective in giving an overall idea about the relative importance of cryptocurrencies in the whole considered period (the network evolution across weekly time windows will be addressed later).

**Figure 2:**
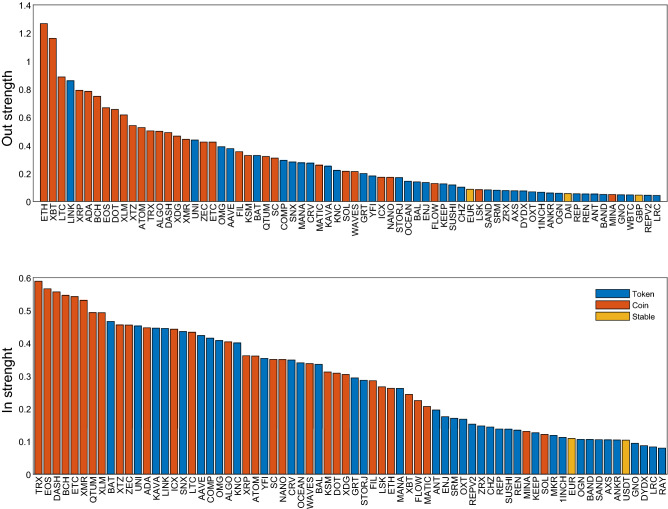
The most important nodes in the network of pairwise Granger causality. Top panel shows the total out strength for the first 70 most influential cryptocurrency, cumulated over the considered two years period. In the same fashion, the bottom panel depicts the in strength of the first 70 most influenced nodes, cumulated over the 104 weekly windows, thus indicating the most influenced nodes of the network. Yellow bars indicate stable-coins, red bars coins and blue bars tokens.

To see more in detail which cryptocurrencies transfer the most information to the rest of the system and which receive the most information from the others, it is useful to look at the ranking of the average in strength and out strength. These information are shown, respectively, in the top and bottom panels of Fig. 2. The different classes of cryptocurrencies - token, coin and stable coins - are represented using different colors. As can be seen in the top panel, the most influential cryptocurrencies belong to the class “coin” (the ones that have their own blockchain, colored in red). Unsurprisingly, the most influential coins are the eldest and most capitalized, such as Bitcoin (XBT), Ethereum (ETH), and Litecoin (LTC). Among the topmost influenced elements (bottom) we find younger and less capitalized “coins”, such as Tron (TRX), EOSIO (EOS) and Dash (DASH). Stable coins seem to play a marginal role in the pairwise description of the network, both for transferred (EUR, DAI, GBP) and received information (EUR, USD).

**Figure 3:**
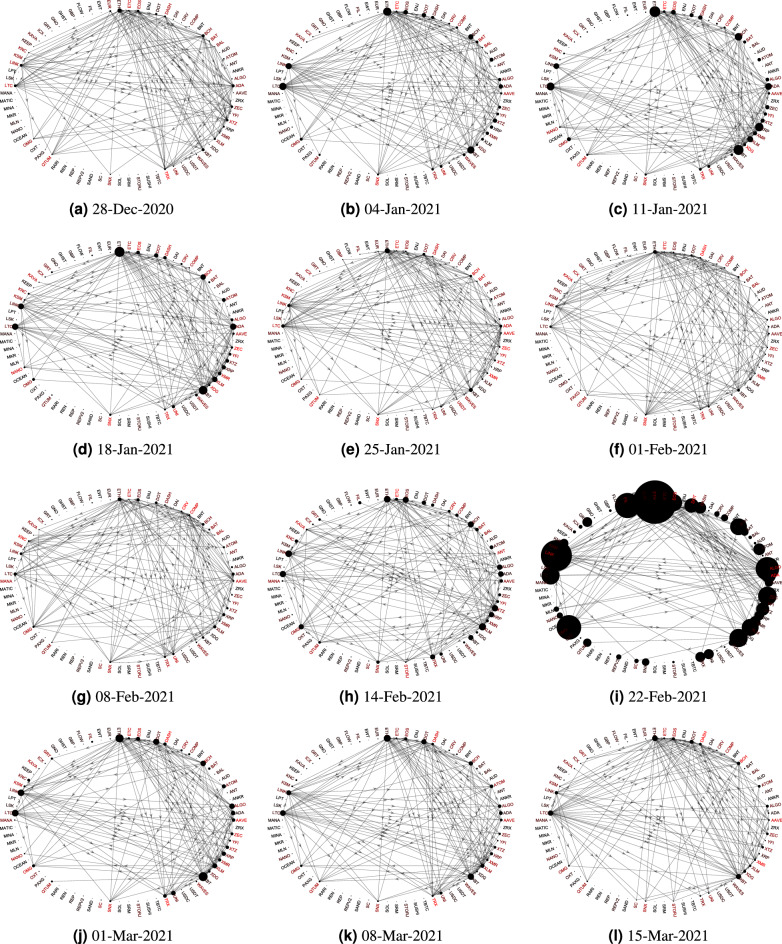
The USD network of pairwise Granger causality for weekly time windows. The size of the nodes represents the total out strength of that node in that window or, in other words, how much that node influences the network, while the label color (from black to red) is the total in strength, that is how much that node is influenced by the rest of the network.

To get a visual hint of how the information flow changes over time, in Fig. 3 we depict the structure of the USD network in several weekly windows (for a period going from 28 Dec, 2020 to 22 Mar, 2021), selected for being stable windows or characterized by the presence of crashes or turbulence. For example, an anomalous activity involving several currencies with high out strength (biggest nodes) can be appreciated in the week starting on 22 Feb, 2021, while during the majority of the other weeks the information flow is visibly much lower. It is worth to notice that the number of nodes in the network slightly increases for subsequent windows, since new cryptocurrencies enter in the market/dataset.

In this regard, it is interesting to investigate if there is some correlation between the age of the cryptocurrencies (i.e. the number of time windows in the USD dataset where it is present) and their in and out strength. As can be seen in the first row of Table 1, actually it seems that such a correlation does exist and is also quite consistent (expecially for the out strength).

**Table 1:** Table shows the Spearman correlation $\rho$ between the in strength and the out strength of a fiat with its “age” (that is, the number of windows in which it was active in the dataset). Two asterisks indicate that $\rho$ is significant at $p<0.01$, one asterisk at $p<0.05$.

Currency	In-strenght	Out-strenght
USD	$0.304^{**}$	$0.442^{**}$
EUR	$0.246^{*}$	$0.400^{**}$
XBT	0.015	0.051
ETH	$-0.152$	$-0.076$

Repeating the same procedure also for the other datasets (where the cryptos are exchanged in different fiats, i.e. EUR, Bitcoin or Ethereum), it results that the correlation stays quite high only when EUR is adopted as fiat (second row in Table 1) while it rapidly disappears for the other fiat currencies (third and fourth rows). This can be explained by the fact that when a given fiat is firstly adopted for the exchange, it is still quite unpopular, and then its connections with the rest of the system from the point of view of Granger causality are still weak. Conversely, as time passed, its importance grows and this is reflected in the values of information flow.

**Figure 4:**
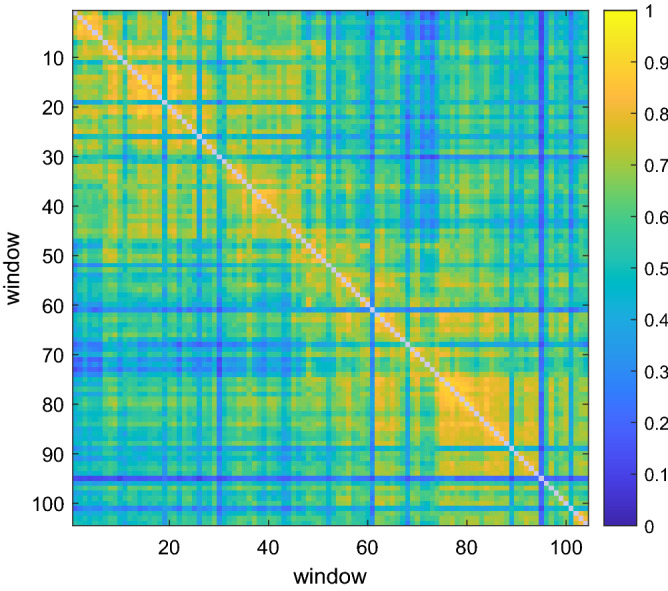
The Pearson correlation between adjacency matrices at different time windows. The element $c_{ij} = \rho (A^i,A^j)$ of the matrix is the Pearson correlation between the vectorized adjacency matrices at windows *i* and *j*. High values of $c_{ij}$ indicate that the structure of the network at windows *i* and *j* is very similar. Diagonal elements, indicating self-correlation, are colored in gray.

Finally, it is also interesting to look at the correlation between pairs of USD networks of Granger causality as they appear in any couple of windows *h* and *k*. To this aim, in Fig. 4 we report the Pearson correlations $\rho (A^{h},A^{k})$ between the adjacency matrices $A^{h}$ and $A^{k}$, for each $h,k=1,2,\ldots ,104$. Correlation $\rho$ can be seen as a measure of similarity between networks: high values of $\rho$ in two different windows indicate that, on average, the information flows between the cryptocurrencies are similar in the corresponding two periods. If we look at values close to the main diagonal, we see that for consecutive windows the correlation is very high, often above 0.9; also, inspecting windows more separate in time (that is, looking at values far from the main diagonal) correlation still remains quite high, indicating that the structure of the Granger causality network is quite stable or, in other words, that the information flow between the various nodes does not change much over time.

Analogous correlation matrices calculated for the other datasets (EUR, XBT and ETH) are reported for completeness in Fig. 9 in Section “Results for other fiat” (of course, as expected for the reasons explained above, correlations between networks traded in XBT and ETH are much less significant with respect to EUR and USD).

Summarizing the results obtained with the Granger pairwise analysis, we can say that, on one hand, as one could expect, for a specific time window nodes influence/get influenced each other according to their out/in strength. On the other hand, the structure of the network stays quite stable over consecutive time windows, since the in/out strength values of nodes remain comparable. In this respect, even if in correspondence of important events or shock in the market the structure of the network seems to change, increasing the number of links and their weight, when the effect of those events vanishes the network come back to its original state.

### O-information

Let us now describe the results of the analysis of the USD dataset from the point of view of high-order dependencies, using dynamical O-information $d\Omega _{\varvec{\tilde{X}}\rightarrow y}$ defined in the “Methods” section. This quantity is a signed metric representing the informational character of the high-order dependencies between the source variables’ past states $\varvec{\tilde{X}}$ and the future states of the target *y*. For each cryptocurrency treated as a target variable, the goal of the analysis is to find the multiplets of variables whose past states $\varvec{\tilde{X}}$ convey the most redundant and synergistic information (occurring for highest and lowest values of $d\Omega _{\varvec{\tilde{X}}\rightarrow y}$, respectively) with the future states *y* of the target variable.

**Figure 5:**
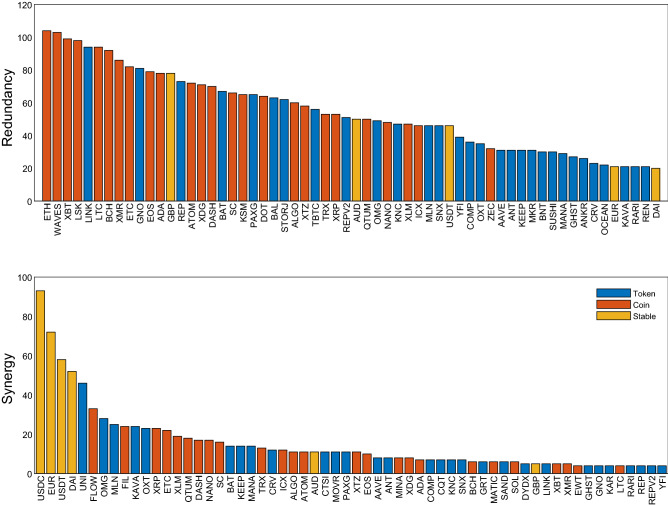
Most influential nodes for synergy and redundancy in the USD dataset. The bar plot counts the number of times a cryptocurrency is found in the best multiplets built as explained in the text. Yellow bars indicates stable-coins, red bars coins and blue bars tokens. Only the first 60 elements are shown.

A first interesting observation can be made about Fig. 5, where it is shown the ranking of the presence of each class of cryptocurrency in the best multiplets, both for redundancy and synergy. Here, on the contrary of the pairwise case, one can observe that stable coins are the variables which belong to the highest number of synergistic information circuits, hence they play a major role for high order effects.

**Figure 6:**
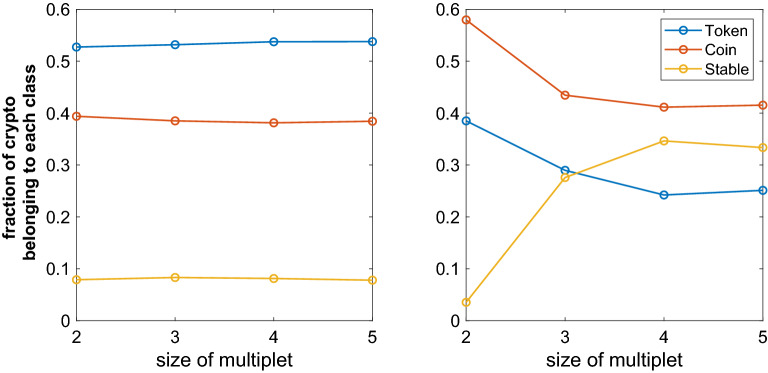
We depict the typical fraction of coin, token and stable-coin in the optimal multiplets $\varvec{\tilde{X}}^n$ as a function of their size *n*, for both redundancy (left) and synergy (right). In the redundant case, these weights are similar to the original dataset (composed of 60 tokens, 33 coins and 6 stable-coins), while for synergistic multiplets coins are the most common and the stable-coins are much more present.

We get other insights by observing the composition of the best multiplets as function of their size. In Fig. 6 we report the typical fraction of elements belonging to the 3 different classes of cryptocurrencies for both redundancy (left panel) and synergy (right panel). Concerning the redundant multiplets, the composition do not depend on the size but only on the class. The highest fraction of elements in these multiplets are tokens, which are also the most common in the original dataset (60/99): high redundancy can be explained from the fact that these cryptocurrency don’t have a own blockchain, but are built on top of another crypto-coin. Notice that the lowest fraction are stable coins, which are the least common in the dataset (6/99).

The situation is very different for the synergistic multiplets (right panel), where the size plays a greater role: actually, the fraction of stable coins increases with the size, while that of the other classes tends to decrease. Interestingly, from size 3 onwards the best multiplets are populated much more often by coins and, in particular, by the stable coins, even if they are very few in the dataset.

These findings are quite surprising since stable coins have a rather marginal activity in the pairwise Granger causality, as can be seen in Fig. 2. This result confirms that taking into account high-order dependencies through O-information provides insights that cannot be retrieved with a pairwise analysis.

### Complementarity of pairwise and high-order descriptions

**Figure 7:**
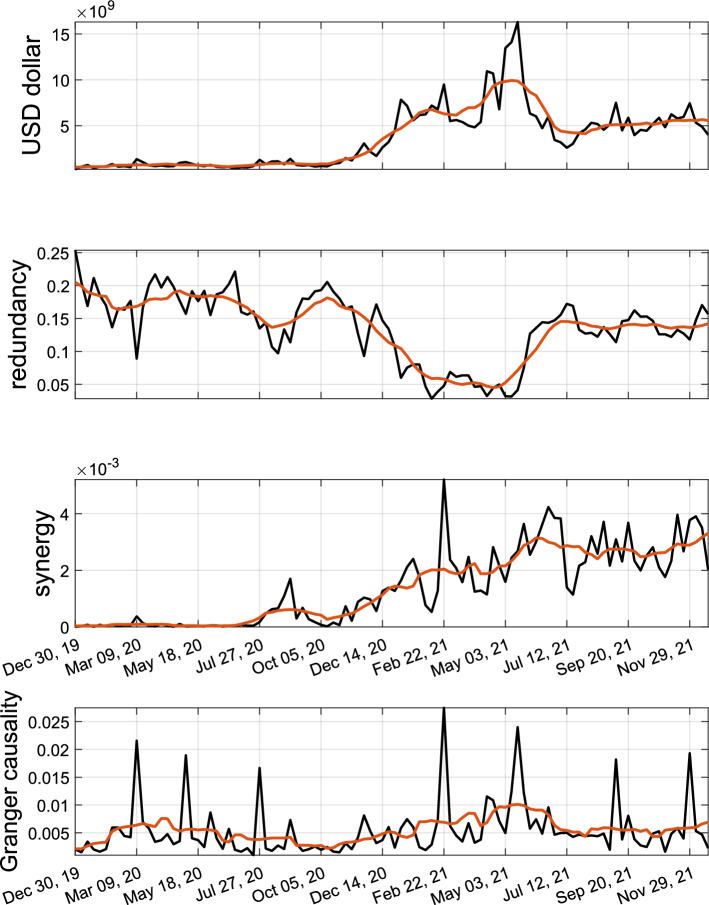
From top to bottom: the total volume (in US dollar) exchanged in each time window; the average value of redundancy and synergy and the average value of Granger causality $\overline{\mathcal {F}}$ calculated for each time window. The 10-weeks moving average of the signal is represented in red.

In order to better highlight the complementarity between the pairwise and the high-order descriptions, in Fig. 7 we make a comparison between the average values of the pairwise and high-order indicators analyzed so far for the USD dataset. The second and third panel (from the top) address the O-information, showing the values of $d\Omega _{\varvec{\tilde{X}^5}\rightarrow y}$ averaged over each target as function of the 104 weekly windows, for redundancy and synergy respectively. In the bottom panel, we depict the analogous time behavior of Granger causality $\mathcal {F}_{X\rightarrow Y}$ averaged over all the possible pairs. Finally, in the top panel, we show the behavior of total exchanges in US dollars, which can be meant as the total volume of transactions which have been performed during each weekly window.

It is quite clear the presence of three successive stages characterized by a different behaviour of the O-information indicators: (i) the first phase, from January to December 2020, shows high redundancy of the market and low synergy; (ii) the second phase, from January to June 2021, is characterized by a fall of the redundancy and a rise of the synergy; (iii) finally, a third phase can be located between July and December 2021 and is characterized by a stationary dynamics of both synergy and redundancy, which stay relatively high. Since synergy may be interpreted as an indicator of the system’s complexity, this result suggests that, during the first semester of 2021, the cryptocurrencies network has undergone a transition toward a more complex dynamical landscape. The transition seems to be triggered by the large volume of transactions occurred in the first semester of 2021.

On the other hand, the behaviour of the Granger causality seems insensitive to such a transition, remaining quite homogeneous during the whole considered period, with the exception of sudden peaks in correspondence of important events - like Covid turbulence in 2020, days of sudden rise in prices etc. Notice, in particular, the peak of 22 Feb 2021, which also appears in the synergy panel and coincides with the first minimum of redundancy. It could be related with the week of anomalous activity already observed in Fig. 3, maybe the effect of some market movements and announcements of Elon Musk about cryptos and their adoption/dismiss as payment method - as reported by financial press (see for example^26^).

**Figure 8:**
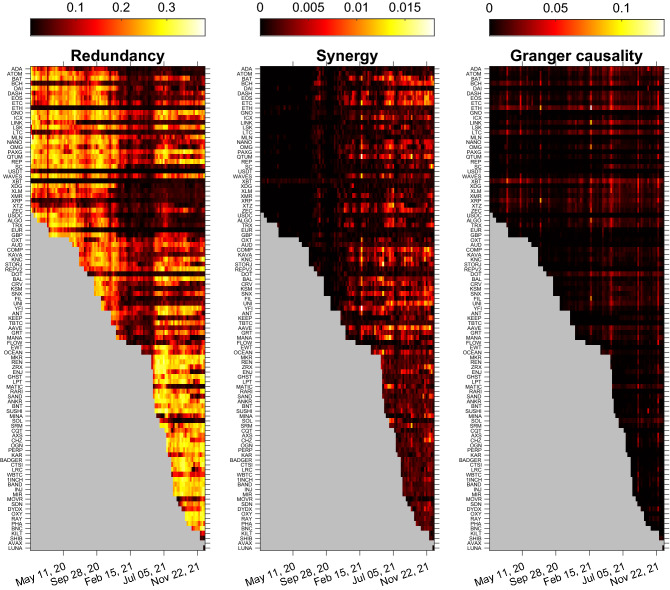
The redundant (left) and synergistic (center) dynamical O-information toward various targets, where each point represents the value of $d\Omega _{{\tilde{X}^5}\rightarrow y}$ of the best multiplet of sources ${\tilde{X}^5}$ that conveys the highest and lowest O-information toward the target *y*. We also depict the out strength Granger causality (right) for each weekly window. Regions in grey represents windows where that cryptocurrency was not yet listed.

A global perspective about the time behaviour of both the O-information and pairwise indicators for each cryptocurrency of the USD dataset can be appreciated in Fig. 8, where we report the same quantities of the previous figure but without averaging over all the crypto. In particular, in the left and central panel it is shown the redundancy and synergistic value of dynamical O-information from the best multiplet, respectively. In the right panel, it is shown the Granger causality out strength of each crypto for each weekly time window. Notice that in such a visualization it becomes clear that only about thirty cryptos (the first from the top) are always present in the dataset for the whole considered time period: the others, ordered according to their age, show an increasing number of weekly windows (colored in gray) in which they are not yet listed.

Focusing on the highest and lowest values of redundancy and synergy, a behaviour analogous to that one observed in Fig. 7 can be easily detected also at this disaggregated level, and the same holds for the Granger causality, thus further confirming the complementarity of the pairwise and the high-order approach.

## Conclusions

In this work, we have analysed the logarithmic USD price returns, building a network of traded cryptocurrency by defining the Granger causality between each pair of cryptocurrencies in the years 2020 and 2021 as links. We used both pairwise and high order statistical dependencies, as measured by Granger causality and O-information, with a dataset consisting of two classes of currencies (coins and tokens) and six stable coins, for a total of 99 time series.

We found that, in terms of Granger causality, the network structure is rather stable across time, referring to weekly time windows in the considered period since, according to our analysis, the set of most influential cryptocoins presents small changes over time. The structure of the network may change due to relevant events or shocks within a specific period, but in a short time it tends to restore its original condition. In the present work we are mainly interested in the stability of the newtork on a global scale. It is worth mentioning that recently several papers dealt with the investigation of time stability of specific links, using network measures such as weighted network liquidity^27^ or assortativity metrics^28–32^; the analysis of local time stability of the cryptocurrencies network is matter for further research.

Turning to consider high-order dependencies, we have analysed how multiplets of cryptocurrencies carry redundant and synergistic information toward the future states of other variables. In synergistic multiplets, as the size of the multiplets increases, the same does the fraction of stable coin on it, despite their marginal role pointed out by the pairwise analysis. On the other hand, in the redundant case, stablecoins fraction is less than 10% and the multiplets composition does not vary while increasing their size.

We obtained other insights by looking at the temporal trends of the pairwise and high-order indicators. In particular, we compared Granger causality, redundancy and synergy with the time evolution of the total amount of exchanges in US dollars, and we found three successive stages characterized by different behaviour of these indicators. The first period, between January to December 2020, is characterized by high redundancy and low synergy among cryptocurrencies. Both change directions on the second phase, starting from January to June 2021, where redundancy fall and synergy rise up. At the last phase, located between July and December 2021, both indicators maintains a stationary dynamics.

These results suggest that pairwise and high-order descriptions of complex financial systems provide complementary information, thus, considered together, they represent a very promising tool for the analysis of cryptocurrency trading networks or similar ones.

Conclusively, considering the amount of resources and the relevance of trading activities related to cryptocurrencies, the analysis of their dynamics is a relevant field of investigation aimed at stabilizing their markets. At the end of the day, cryptocurrencies are not financial instruments with an intrinsic value representing productive assets. They have been conceived as mediums of exchange, in markets where their quotation is totally self-referenced and driven by their adoption in transactions (often of unknown type and content).

Nonetheless, the attracting appeal of positive price jumps and bubbles induces investors to underestimate potential losses. Our analysis of the network of cryptocurrencies might be useful to unveil that while drivers of instability cannot be removed in general, specially in the absence of intrinsic – fundamental – values, quotations and prices become extremely risky and unpredictable. This, on a normative side, could inspire policies aimed at fostering stability of markets, anchored more to produced values than to financial speculation.

Matter of factly, some attempts have been done to create a formal market structure for some crypto (i.e. lending platforms such as CoinLoan or Hodlnaut, Binance and Crypto.com as an example of crypto-debit payments card and even Sandbox virual world, where each player can buy objects using cryptocoins and even earning coins by playing the game). The need for a more “readable” orientation, aimed at providing priors on the market configuration and on its physiology, shows that despite a financial artefact may reveal appealing in block-chain transactions, it might be perceived as dangerous and too risky.

Our analysis goes to the direction of investigating and understanding what moves the dynamics of such highly volatile crypto currencies, which can be useful from a policy standpoint in order to foster stability and prevent the destruction of relevant financial values.

## Methods

### Pairwise Granger Causality

In this section, we describe how to implement Granger Causality between pair of variables in the context of linear autoregressive models.

Let us consider two stationary time series $X_{t}$ and $Y_{t}$. Under suitable conditions^33^, the target series *Y* can be written as a weighted sum of its past states and an error term $\epsilon '_t$ (reduced model); similarly, a second model can be built by adding the past states of the source variable (full model):

1 $$\begin{aligned} Y_{t}&= \sum _{m=1}^p a_{m}Y_{t-m} + \epsilon '_t \end{aligned}$$

2 $$\begin{aligned} Y_{t}&= \sum _{m=1}^p a_{m}Y_{t-m} + \sum _{m=1}^q b_{m}X_{t-m} + \epsilon _t~ \end{aligned}$$

In other words, in the full model, the future states of the target are predicted using its previous *p* past states and the previous *q* states of the source, while in the reduced model only the target is involved.

The goodness of these models can be quantified by the variance of the error term of the full model $\sigma ^2_f = \langle \epsilon ^2_t\rangle$ and of the reduced model $\sigma _{r}^2 = \langle \epsilon ^{'2}_t\rangle$, where $\langle \cdot \rangle$ indicates the expected value. To test if the full model improves the predictability of the target, we use the statistics

3 $$\begin{aligned} \mathcal {F}_{X\rightarrow Y} = \ln \frac{\sigma _{r}^2}{\sigma ^2_f}~. \end{aligned}$$

This choice has a number of useful properties^34^, remarkably the invariance under scaling transformation of the original time series and the asymptotic chi-square distribution under the null hypothesis $\mathcal {F}_{X\rightarrow Y} =0$. To build a bridge between Granger causality and the information-theoretical methods for analyzing high-order dependencies of the next section, we can consider the equivalence between Granger causality and transfer entropy $\mathcal {T}$ under the Gaussian approximation^24^.

Transfer entropy is a model-free version of Granger causality. Considering the source states $\varvec{X}^{-}_t = (X_{t-1},X_{t-2},\ldots ,X_{t-p})$, at varying *t*, as realizations of a stochastic variable $\varvec{X}^-$, and analogously $\varvec{Y}_t^-= (Y_{t-1},Y_{t-2},\ldots ,Y_{t-p})$ as realizations of the target state variable $\varvec{Y}^-$, $Y_{t}$ as realizations of the future of the target y, considered as a stochastic variable, the transfer entropy is defined as the mutual information between source and target variable conditioned over the target past states:

4 $$\begin{aligned} \mathcal {T}_{X\rightarrow Y} = I(y ; \varvec{X}^-|\varvec{Y}^-) \end{aligned}$$

The equivalence reads:

5 $$\begin{aligned} \mathcal {F}_{X\rightarrow Y} = 2 \mathcal {T}_{X\rightarrow Y} \end{aligned}$$

The pairwise Granger causality has been computed for each pair using the MATLAB toolbox MVGC^35^. The result of Granger causality produces an adjacency matrix $A = \{a_{ij}\}$, where the element $a_{ij}$ represents the Granger Causality from the cryptocoin *i* toward the cryptocoin *j*, which can be used as weighted links for our directed Cryptocurrencies Trading Network.

To find the most influencing and influenced nodes, one may define the quantities out strength and in strength:

6 $$\begin{aligned} k^{\mathrm{out}}_i= & {} \sum _{j\ne i} a_{ij} \end{aligned}$$

7 $$\begin{aligned} k^{\mathrm{in}}_i= & {} \sum _{j\ne i} a_{ji} \end{aligned}$$

### O-information

We now describe how to study high-order dependencies in a collection of random variables $\varvec{X}^n = (X_1,X_2,\ldots ,X_n)$. The first step is to extend the concept of mutual information to the multivariate case: a popular way to do it is through *total correlation* (also known as multi-information)^36^, which quantifies the amount of high-order constraints and then is a measure of redundant information. In terms of information-theoretical quantities, it can be expressed as:

8 $$\begin{aligned} \mathcal {T}(\varvec{X}^n) = \sum _{k=1}^n H(X_k) - H(\varvec{X}^n), \end{aligned}$$

where $H(X_k)$ and $H(\varvec{X}^n)$ are the Shannon entropies, which quantify the uncertainty of the k-th variable and of the whole system, respectively. Another useful way of looking at this quantity is as a measure of the “statistical constraints” acting on $X_k$: when *H* is low, the system explores more frequently small regions of the phase space and, in this sense, the constraints are high. Conversely, when *H* is maximum (a condition that occurs when the distribution is uniform), then there is the minimum possible amount of constraints acting on $X_k$.

Another popular extension of mutual information is the *dual total correlation*^37^

9 $$\begin{aligned} \mathcal {D}(\varvec{X}^n) = H(\varvec{X}^n) - \sum _{k=1}^n H(X_k\vert \varvec{X}^n_{-k}) \end{aligned}$$

where $\varvec{X}^n_{-k}$ represents all the system minus the k-th variable. $\mathcal {D}(\varvec{X}^n)$ may be interpreted as the amount of uncertainty that can be explained only by observing more than one variable at once: for this reason, dual total correlation is a measure of the synergy present in the system at global level. Crucially, it can be easily shown that both $\mathcal {T}(\varvec{X}^n)$ and $\mathcal {D}(\varvec{X}^n)$ are nonnegative^38^ and then can be used as proper measures of redundancy and synergy.

The following quantity, called O-information^12^, represents the balance between the redundant and the synergistic dependencies in $\varvec{X}^n$:

10 $$\begin{aligned} \Omega (\varvec{X}^n)&= \mathcal {T}(\varvec{X}^n) - \mathcal {D}(\varvec{X}^n) \end{aligned}$$

11 $$\begin{aligned}&=(n-2)H(\varvec{X}^n)+ \sum _{k=1}^n \big [H(X_k) - H(\varvec{X}^n_{-k}) \big ]. \end{aligned}$$

Accordingly, if this quantity is greater than 0, we say that the system is redundancy-dominated, whilst if $\Omega < 0$ the system is synergy-dominated.

Now, we introduce a target variable *y* to study whether the high-order dependencies between a multiplet of *n* variables $\varvec{X}^n$ with *y* are redundant or synergistic. We can express the O-information of the system jointly formed by the multiplet and the target as follows

12 $$\begin{aligned} \Omega (\varvec{X}^n\cup y) = \Omega (\varvec{X}^n) + \Delta ^y \end{aligned}$$

where $\Delta ^y$ the contribution of a variable to the O-information and reads:

13 $$\begin{aligned} \Delta ^y = (1-n)I(y;\varvec{X}^n) + \sum _{k=1}^n I(y;\varvec{X}^n_{-k}), \end{aligned}$$

where $I(y;\varvec{X}^n) = H(y) + H(\varvec{X}^n) - H(y;\varvec{X}^n)$ denotes mutual information.

Now we turn to consider *n* time series $\{X_{i,t}\}$ and a target $\{Y_{t}\}$, where $t=1,\ldots ,T$ and $i=1,\ldots ,n$; the informational character of the information flow from the multiplet of X variables (considered as sources) to the target, can be assessed introducing stochastic variables $\varvec{X}^{n-} = (X_1^-,X_2^-,\ldots ,X_n^-)$, $\varvec{Y}^{-}$ and *y* representing the sources, the target and the future of the target respectively, whose realizations can be obtained from the samples multivariate time series.

Hence, the dynamical version of the O-information, introduced in^39^ for the analysis of neural signals, reads

14 $$\begin{aligned} d\Omega ^y(\varvec{X}^n) = (1-n)I(y;\varvec{X}^{n-}|\varvec{Y}^-) + \sum _{k=1}^n I(y;\varvec{X}^{n-}_{-k}|\varvec{Y}^-). \end{aligned}$$

This quantity can be also thought as a high-order version of the Granger causality and its sign has the same interpretation of the O-information in terms of redundancy and synergy.

In order to analyse the dataset, we proceed as follows. First, we fixed a target variable *y*; then, we searched among the remaining $n-1$ variables for the pair that maximize and minimize the quantity

15 $$\begin{aligned} d\Omega ^y_{\text {red}}(\varvec{X}^2)= & {} \max _{i,j} d\Omega ^y(\{X_i X_j\}) \end{aligned}$$

16 $$\begin{aligned} d\Omega ^y_{\text {syn}}(\varvec{X}^2)= & {} \min _{i,j} d\Omega ^y(\{X_i X_j\}). \end{aligned}$$

Then, $d\Omega ^y_{\text {red}}(\varvec{X}^2)$ and $d\Omega ^y_{\text {syn}}(\varvec{X}^2)$ is the highest and lowest dynamic O-information to the target *y* from the best multiplets ${\varvec{\tilde{X}^{2}}} = \{{X}_{i}^{*} {X}_{j}^{*}\}$ of size $n=2$. For sizes $n>2$, we used a greedy approach: for $n=3$, we start from the ${\varvec{\tilde{X}^{2}}} = \{X_{i^*} X_{j^*}\}$ found in the previous step, and for the remaining variables $X_k$ we choose the one that maximizes and minimize $d\Omega ^y(\{X_i X_j X_k\})$. We repeated the same procedure for $n=4$ and so on, and we stop at $n=5$.

## Data and preprocessing

The dataset investigated in this study is freely available from Kraken crypto Exchange^40^ and contains traded prices in general between cryptocoins and currencies.

In the following we will use different names for cryptocurrencies according to their own properties. We refer more specific to a Coin to describe a cryptocoin with its blockchain, to a Token to describe a cryptocoin backed by a blockchain of another Coin. Also, we refer as Stablecoin to indicate to a Cryptocoin which has its price “pegged” to the price of another asset (usually the USD change value) and in general to a Fiat currency to any government-issued currency such as Euro, Dollar or others as described in^41^.

Each file of the dataset is organized in “pairs”, where one pair consist of a cryptocurrency traded using a fiat currency, so it reports all trade on a specific market, which collect bid and ask orders on a corresponding order book, from cryptocurrency to fiat currency and vice-versa. Each line refers to a single trade or transaction. Since it is possible to have more than one transaction per second referring to the same timestamp, with different price and volume, they are reported on separate lines.

For each pair, data provided for each trade have the following information:

- *timestamp* represents the time when the trade occurs, with resolution in seconds. It is possible to have more than one trade with the same timestamp, in this case volume and price may be different for each trade;
- *price* is the price at which the cryptocurrency was traded, in terms of the corresponding fiat currency;
- *volume* is the amount of cryptocurrency exchanged on the corresponding trade;

Data were aggregated on the scale of minutes, considering the weighted mean over volume for the minute price of the trade, and the sum of volumes for the total volume exchanged at the current minute time. If there were no trade on a minute, in order to avoid discontinued time series, we hold the previous last price to fill the gap on the time series, setting the corresponding volume to zero. Then, we computed the logarithmic returns of each time series of the price, which will be divided into time series of weekly length, to see how the results vary over time and to take into account effect due to the different volume of transactions over the week (usually smaller volumes are traded during weekends). We divided the considered period in weekly time windows, each starting at 00:00:00 every Monday and terminating at 23:59:59 every Sunday (with the exception of last window because last day available on the dataset was Friday, 31 Dec 2021), thus obtaining 104 windows, each containing a total of 10080 points.

We remark that the analysis is performed just on the time series of logarithmic returns; the time series of volumes are used here only to compare the time evolution of information-theoretical quantities with the total volume of exchanges in US dollars. A cryptocurrency can be traded on the Exchange using more than one fiat, then there is the possibility to have more than one pair for each crypto, one for each available fiat market (i.e. Bitcoin can be traded in EUR, Dollar or other fiat currencies related to the country of the user). Also, the Exchange allowed to use some crypto coins (i.e. Bitcoin, Ethereum) as fiat, probably to speed up buying and selling operations.

Table  2 in section "Data Availability" reports a brief description of all data available on Kraken, grouped by years, number of cryptos and fiat, while Table  3 shows which tickers are included on the dataset and some of their features.

**Table 2:** Number of cryptocoins and fiat available on Kraken during years. At the beginning of the launch of the Kraken platform, there were available only two cryptos (Bitcoin as XBT and Litecoin as LTC) traded using 2 fiat (Euro and Dollar, using EUR and USD as corresponding tickers). As far as the platform grow up, there were listed more cryptos and fiat, up to 2021 when there are more than 90 cryptos available, and traded using 12 fiat. Since 2016, Bitcoin and Ethereum were also used as “fiat”, to simplify the process of buying and selling.

Year	Number of crypto	Number of fiat
2013	2	2
	(XBT, LTC)	(EUR, USD)
2014	2	4
		(+ GBP,JPY only for XBT)
2015	3	5
	(+ ETH)	(+ CAD for XBT and ETH)
2016	9	7
	(+ZEC,XLM,REP,XRP,XDG,ETC)	(+XBT,ETH as fiat)
2017	16	7
	(+ USDT,DASH,XMR,GNO,MLN,EOS,BCH)	
2018	19	7
	(+ ADA,QTUM,XTZ)	
2019	30	10
	(...)	(+ USDT,CHF,DAI)
2020	60	11
	(...)	(+AUD)
2021	99	12
	(...)	(+DOT)

**Table 3:** Tickers description. Each row provides some information on the ticker, such as the name of the coin, the website of the project, its type (COIN for cryptocoin which hold their own blockchain, TOKEN for cryptos built on top of another cryptocoin, fiat for currencies and TOKEN - USD Stablecoin for token hooked to the USD dollar price) and what algorithm it is used as consensus mechanism (for COINS only).

Ticker	Name	Website	Type	Consensus mechanism
1INCH	1inch	https://1inch.io/	TOKEN	
AAVE	Aave	https://aave.com/	TOKEN	
ADA	Cardano	https://cardano.org	COIN	Proof of Stake
ALGO	Algorand	http://algorand.foundation/	COIN	Proof of Stake
ANKR	Ankr	https://www.ankr.com/	TOKEN	
ANT	Aragon	https://aragon.org/	TOKEN	
ATOM	Cosmos	https://cosmos.network/	COIN	Proof of Stake
AVAX	Avalanche	https://avax.network/	COIN	Proof of Stake
AXS	Axie Infinity Shards	https://axieinfinity.com/	TOKEN	
BADGER	Badger DAO	https://app.badger.finance/	TOKEN	
BAL	Balancer	https://balancer.finance/	TOKEN	
BAND	Band Protocol	https://bandprotocol.com/	TOKEN	
BAT	Basic Attention Token	https://basicattentiontoken.org/	TOKEN	
BCH	Bitcoin Cash	http://bch.info/	COIN	Proof of Work
BNC	Bifrost	https://bifrost.finance/	TOKEN	
BNT	Bancor	https://bancor.network/	TOKEN	
CHZ	Chiliz	https://www.chiliz.com/	TOKEN	
COMP	Compound	https://compound.finance/governance/comp	TOKEN	
CQT	Covalent	https://www.covalenthq.com/	TOKEN	
CRV	Curve	https://www.curve.fi/	TOKEN	
CTSI	Cartesi	https://cartesi.io/	TOKEN	
DAI	Dai	http://www.makerdao.com/	TOKEN - USD StableCoin	
DASH	Dash	https://www.dash.org/	COIN	Hybrid - PoW & PoS
DOT	Polkadot	https://polkadot.network/	COIN	Nominated Proof of Stake
DYDX	dYdX	https://dydx.community/	TOKEN	
ENJ	Enjin Coin	https://enjin.io/	TOKEN	
EOS	EOSIO	https://eos.io	COIN	Delegated Proof of Stake
ETC	Ethereum Classic	https://ethereumclassic.org/	COIN	Proof of Work
ETH	Ethereum	https://www.ethereum.org/	COIN	Proof of Work
ETH2.S	Ethereum 2.0 Staking			Proof of Stake
EUR			FIAT	
EWT	Energy Web	https://www.energyweb.org/	COIN	Proof of Authority
FIL	Filecoin	https://filecoin.io/	COIN	Proof-of-Replication and Proof-of-Spacetime
GBP			FIAT	
GHST	Aavegotchi	https://aavegotchi.com/	TOKEN	
GNO	Gnosis	https://gnosis.io/	TOKEN	
GRT	The Graph	https://thegraph.com/	TOKEN	
ICX	Icon	https://icon.community/	COIN	Proof of Stake
INJ	Injective	https://injective.com/	TOKEN	
KAR	Karura	http://karura.network/	TOKEN	
KAVA	Kava	https://www.kava.io/	TOKEN	
KEEP	Keep Network	https://keep.network/	TOKEN	
KILT	Kilt Protocol	https://kilt.io/	TOKEN	
KNC	Kyber Network	https://kyber.network/	TOKEN	
KSM	Kusama	https://kusama.network/	COIN	Nominated Proof of Stake
LINK	Chainlin	https://chain.link/	TOKEN	
LPT	Livepeer	https://livepeer.org/	TOKEN	
LRC	Loopring	https://loopring.org/	TOKEN	
LSK	Lisk	https://lisk.com/	COIN	Delegated Proof of Stake
LTC	Litecoin	https://litecoin.org/	COIN	Proof of Work
LUNA	Terra	https://terra.money/	COIN	Proof of Stake
MANA	Decentraland	https://decentraland.org/	TOKEN	
MATIC	Polygon	https://polygon.technology/	COIN	Proof of Stake
MINA	Mina	https://minaprotocol.com/	COIN	Zk-SNARK
MIR	Mirror Protocol	https://mirror.finance/	TOKEN	
MKR	Maker	https://makerdao.com/	TOKEN	
MLN	Enzyme Finance	https://enzyme.finance/	TOKEN	
MOVR	Moonriver	https://moonbeam.network/networks/moonriver/	TOKEN	
NANO	Nano	https://nano.org/en	COIN	Delegated Proof of Stake
OCEAN	Ocean	https://oceanprotocol.com/	TOKEN	
OGN	Origin	https://www.originprotocol.com/	TOKEN	
OMG	OMG Network	https://omg.network/	TOKEN	
OXT	Orchid	https://www.orchid.com/	TOKEN	
OXY	Oxygen	https://www.oxygen.org/	TOKEN	
PAXG	PAX Gold	https://www.paxos.com/paxgold/	TOKEN	
PERP	Perpetual Protocol	https://perp.com/	TOKEN	
PHA	Phala	https://phala.network/	TOKEN	
QTUM	Qtum	https://qtum.org/	COIN	Mutualized Proof of Stake
RARI	Rarible	https://app.rarible.com/rari	TOKEN	
RAY	Raydium	https://raydium.io/	TOKEN	
REN	Ren	https://renproject.io/	TOKEN	
REP	Augur	http://www.augur.net/	TOKEN	
REPV2	Augur v2	http://www.augur.net/	TOKEN	
SAND	Sandbox	https://www.sandbox.game/en/	TOKEN	
SC	Siacoin	https://sia.tech/	COIN	Proof of Work
SDN	Shiden	https://shiden.astar.network/	TOKEN	
SHIB	Shiba Inu	https://shibatoken.com/	TOKEN	
SNX	Synthetix	https://www.synthetix.io/	TOKEN	
SOL	Solana	https://solana.com/	COIN	Proof of Stake
SRM	Serum	https://projectserum.com/	TOKEN	
STORJ	Storj	https://storj.io/	TOKEN	
SUSHI	Sushi	https://sushi.com/	TOKEN	
TBTC	tBTC	https://tbtc.network/	TOKEN	
TRX	Tron	https://tron.network/	COIN	Delegated Proof of Stake
UNI	Uniswap	https://uniswap.org/blog/uni/	TOKEN	
USD			FIAT	
USDC	USD Coin	https://www.centre.io/usdc	TOKEN - USD StableCoin	
USDT	Tether	https://tether.to/	TOKEN - USD StableCoin	
WAVES	Waves	https://waves.tech/	COIN	Leased Proof of Stake
WBTC	Wrapped Bitcoin	https://wbtc.network/	TOKEN	LpoS
XBT	Bitcoin	https://bitcoin.org/	COIN	Proof of Work
XDG	Dogecoin	http://dogecoin.com/	COIN	Proof of Work
XLM	Stellar	https://www.stellar.org/	COIN	Stellar Consensus Protocol
XMR	Monero	https://www.getmonero.org/	COIN	Proof of Work
XRP	Ripple	https://ripple.com/	COIN	Ripple consensus
XTZ	Tezos	https://www.tezos.com/	COIN	Liquid Proof of Stake
YFI	yEarn	https://yearn.finance/	TOKEN	
ZEC	Zcash	https://z.cash/	COIN	Zk-SNARK
ZRX	0x	https://0x.org/	TOKEN

**Table 4:** Data coverage on 2020 (starting on Monday 30/12/2019) and 2021. Rows are cryptocoins, columns correspond to fiat coin used to trade. The crypto column reports the Ticker used to list the cryptocoin on the Kraken Exchange. Firstday column show when the cryptopair appear for the first time (on the 2020 or 2021) using the format yyymmdd. For each fiat, are shown the number of days available for the crypto-fiat pair in 2020 and in 2021.

crypto	firstday	2020													2021												
		EUR	USD	GBP	CAD	AUD	CHF	DAI	JPY	USDT	USDC	XBT	ETH	DOT	EUR	USD	GBP	CAD	AUD	CHF	DAI	JPY	USDT	USDC	XBT	ETH	DOT
1INCH	20210810														144	144											
AAVE	20201215	17	17	17								17	17		365	365	365		345						365	365	
ADA	20191230	368	368							78		368	368		365	365	345		345				365		365	365	
ALGO	20200122	345	345									346	345		365	365	345								365	365	
ANKR	20210524														222	222	222								222		
ANT	20201124	38	38									38	38		365	365									365	365	
ATOM	20191230	368	368									368	368		365	365	345		345						365	365	
AUD	20200616		199						199							365						363					
AVAX	20211221														11	11											
AXS	20210713														172	172											
BADGER	20210803														151	151											
BAL	20200917	106	106									106	106		365	365									365	365	
BAND	20210810														144	144											
BAT	20191230	368	368									368	368		365	365									365	365	
BCH	20200616	368	368	246		199			71	246		368	246		365	365	365		365			365	365		365	365	
BNC	20211026														67	67											
BNT	20210524														222	222	222								222		
CHZ	20210713														172	172											
COMP	20200715	170	170									170	170		365	365									365	365	
CQT	20210706														179	179											
CRV	20200917	106	106									106	106		365	365									365	365	
CTSI	20210803														151	151											
DAI	20191230	368	368							368					365	365							365				
DASH	20191230	368	368									368			365	365									365		
DOT	20201015	136	136							78		136	136		365	365	345		345				365		365	365	
DYDX	20210914														109	109											
ENJ	20210520														226	226	225								226		
EOS	20201015	368	368							78		368	368		365	365							365		365	365	
ETC	20191230	368	368									368	368		365	365									365	365	
ETH	20191230	368	368	368	368	198	368	368	368	368	359	368			365	365	365	365	365	365	365	365	365	365	365		
ETH2.S	20211001																									92	
EUR	20200617		295	295	295	198	295		295							365	365	365	365	365		365					
EWT	20210401														275	275	275								275		
FIL	20201015	78	78									78	78		365	365	345		345						365	365	
FLOW	20210127														339	339	339								339	339	
GBP	20200312		295													365											
GHST	20210517														229	229	229								229		
GNO	20191230	368	368									368	368		365	365									365	365	
GRT	20201218	14	14									14	14		365	365	345		345						365	365	
ICX	20191230	368	368									368	368		365	365									365	365	
INJ	20210810														144	144											
KAR	20210720														165	165											
KAVA	20200715	170	170									170	170		365	365									365	365	
KEEP	20201124	38	38									38	38		365	365									365	365	
KILT	20211130														32	32											
KNC	20200715	170	170									170	170		365	365									365	365	
KSM	20210121	106	106									106	106		365	365	345		344						365	365	267
LINK	20210121	368	368							78		368	368		365	365	345		345				365		365	365	
LPT	20210520														226	226	226								226		
LRC	20210803														151	151											
LSK	20191230	368	368									368	368		365	365									365	365	
LTC	20191230	368	368	245		198			71	246		368	246		365	365	365		365			365	365		365	365	
LUNA	20211216														16	16											
MANA	20201215	17	17									17	17		365	365									365	365	
MATIC	20210517														229	229	229								229		
MINA	20210601														214	214	214								214		
MIR	20210810														144	144											
MKR	20210514														232	232	232								232		
MLN	20191230	368	368									368	368		365	365									365	365	
MOVR	20210827														127	127											
NANO	20191230	368	368									368	368		365	365									365	365	
OCEAN	20210401														275	275	275								275		
OGN	20210713														172	172											
OMG	20191230	368	368									368	368		365	365									365	365	
OXT	20200602	213	213									213	213		365	365									365	365	
OXY	20210928														95	95											
PAXG	20191230	368	368									368	368		365	365									365	365	
PERP	20210713														172	172											
PHA	20211008														85	85											
QTUM	20191230	368	368									368	368		365	365									365	365	
RARI	20210517														229	229	229								229		
RAY	20210928														95	95											
REN	20210514														232	232	232								232		
REP	20191230	368	368									368	368		365	365									365	365	
REPV2	20200804	150	150									150	150		365	365									365	365	
SAND	20210520														226	226	226								226		
SC	20191230	368	368									368	368		365	365									365	365	
SDN	20210902														121	121											
SHIB	20211130														32	32											
SNX	20200917	106	106									106	106		365	365	345		345						365	365	
SOL	20210617														198	198	198								198		
SRM	20210617														198	198	198								198		
STORJ	20200715	170	170									170	170		365	365									365	365	
SUSHI	20210524														222	222	222								222		
TBTC	20201125	38	37									37	38		365	365									365	365	
TRX	20200305	302	302									302	302		365	365									365	365	
UNI	20201015	78	78									78	78		365	365									365	365	
USD	20200312				295		295		295									365		365		365					
USDC	20200108	359	359							359					365	365	345		345				365				
USDT	20191230	368	368	368	368	198	245		244						365	365	365	365	365	365		365					
WAVES	20191230	368	368									368	368		365	365									365	365	
WBTC	20210825														151	151									129		
XBT	20191230	368	368	368	368	198	368	368	368	368	359				365	365	365	365	365	365	365	365	365	365			
XDG	20191230	368	368									368			365	365							196		365		
XLM	20191230	368	368									368			365	365	345		345						365		
XMR	20191230	368	368									368			365	365									365		
XRP	20191230	368	368	246	368	198			368	246		368	246		365	365	365	365	365			365	365		365	365	
XTZ	20191230	368	368									368	368		365	365	345		345						365	365	
YFI	20201015	78	78									78	78		365	365	345		345						365	365	
ZEC	20191230	368	368									368			365	365									365		
ZRX	20210514														232	232	232								232		

As the cryptocoin market grows, the number of cryptos and fiat listed on Kraken increases to about hundred cryptocoins, traded up to 12 different market, for a total of about 450 crypto-fiat pairs.

In our analysis, we focus on the period 2020-2021 since the number of cryptos is large and the volume of the transaction is also large. It is possible to have an overview of the data related to each cryptos for 2020-2021 on Table 5.

### Results for other fiat

**Figure 9:**
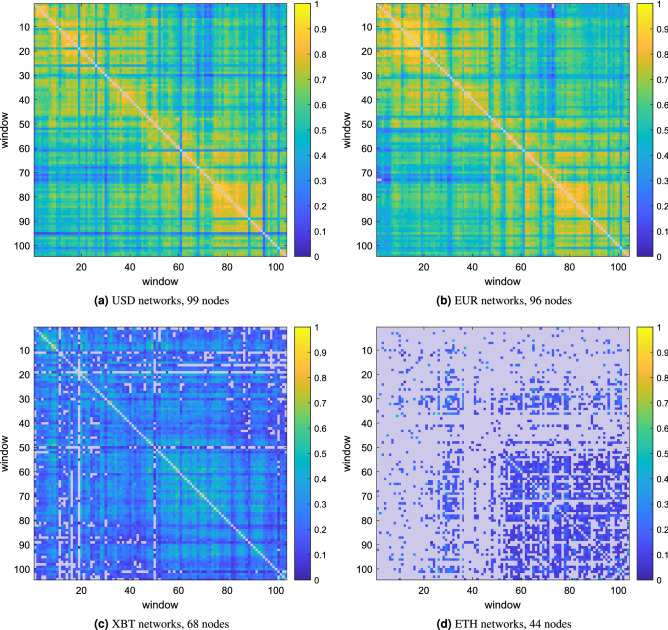
The Pearson correlation between adjacency matrices at different time windows. The element $c_{ij} = \rho (A^i,A^j)$ of each of these matrices is the Pearson correlation between the vectorized adjacency matrices at windows *i* and *j*. High values of $c_{ij}$ indicate that the structure of the network at windows *i* and *j* is very similar. 9b and 9a are the correlation between cryptos networks traded using respectively USD and EUR. As it is possible to see they are quite similar, except for some window where correlation is discontinued on USD. Panels 9c and 9d show the correlation between networks using XBT and ETH as fiat. Despite the number of nodes being similar to USD and EUR networks, the correlation profile is quite low compared to them. Correlations that are not significant ($p > 0.01$), are indicated in gray.

For completeness, in Fig. 9 we show the time correlation matrices obtained by using also the other fiat currencies of the dataset. Concerning the stability of the network, we see that results for EUR 9b are quite similar to USD 9a. On the other hand, in Figs. 9c and d it can be seen that the matrix of correlation for XBT and ETH has low and often non-significant values, indicating that the network is quite unstable.

## Data Availability

The original dataset can be download from the Kraker website^40^. Other mid-computation data can be provided on request from the corresponding author, [T.S.]. In Tables 2, 3 and 5 we provide more details on the global Kraken dataset used in the analysis.
